# Supplementary figures and images for: A Positive Feedback Loop Exists between Estradiol and IL-6 and Contributes to Dermal Fibrosis
Source: Int J Mol Sci. 2024 Jun 30;25(13):7227. doi: 10.3390/ijms25137227 (PMC11241801; doi:10.3390/ijms25137227)

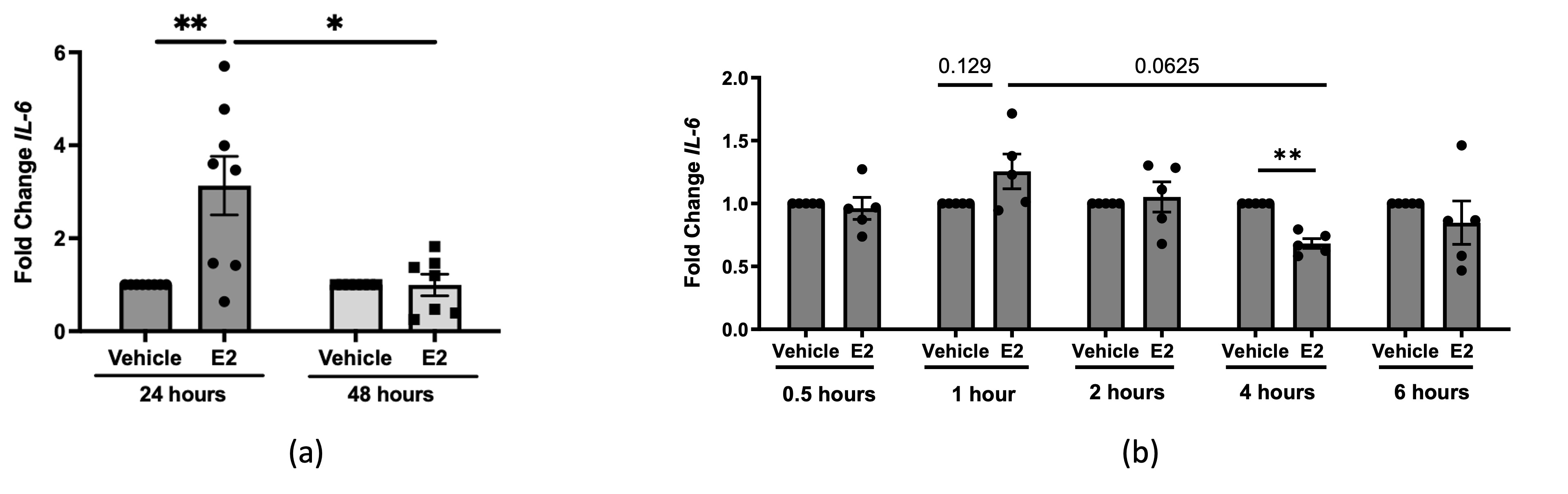

Supplement: Supplementary file 1 [file ijms-25-07227-s001.zip › supplementary figure 1.jpg]

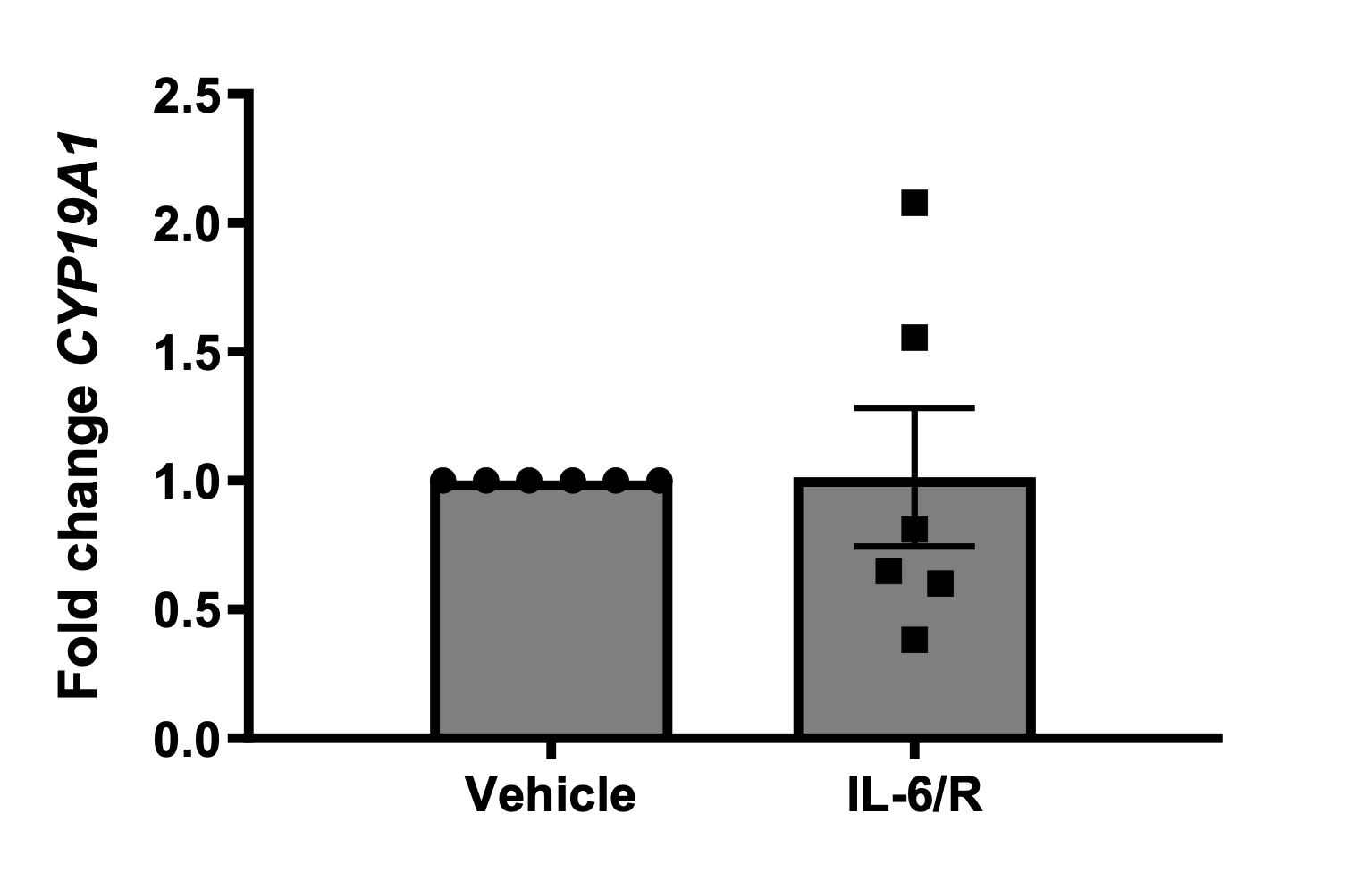

Supplement: Supplementary file 1 [file ijms-25-07227-s001.zip › supplementary figure 2.jpg]

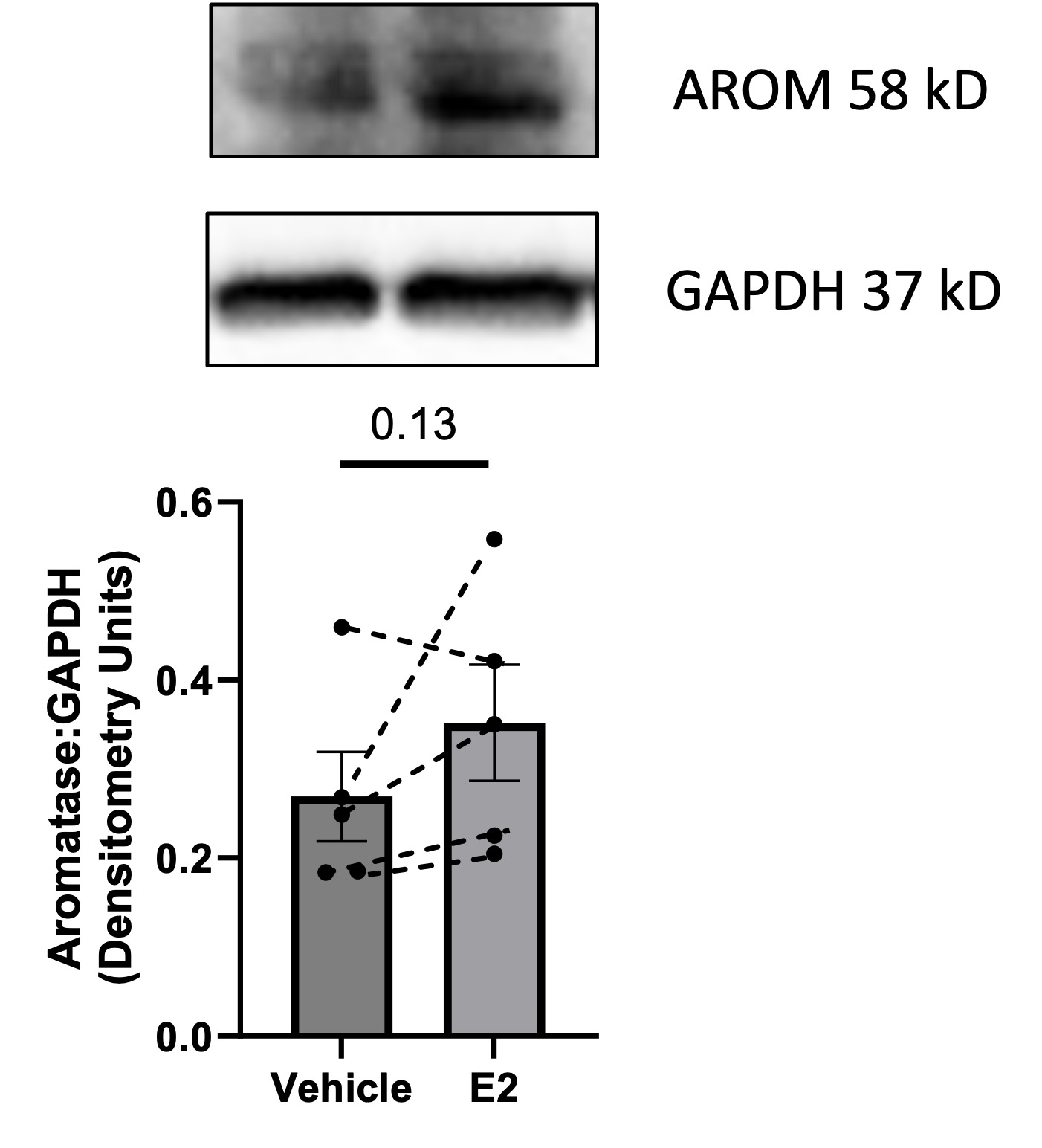

Supplement: Supplementary file 1 [file ijms-25-07227-s001.zip › supplementary figure 3.jpg]

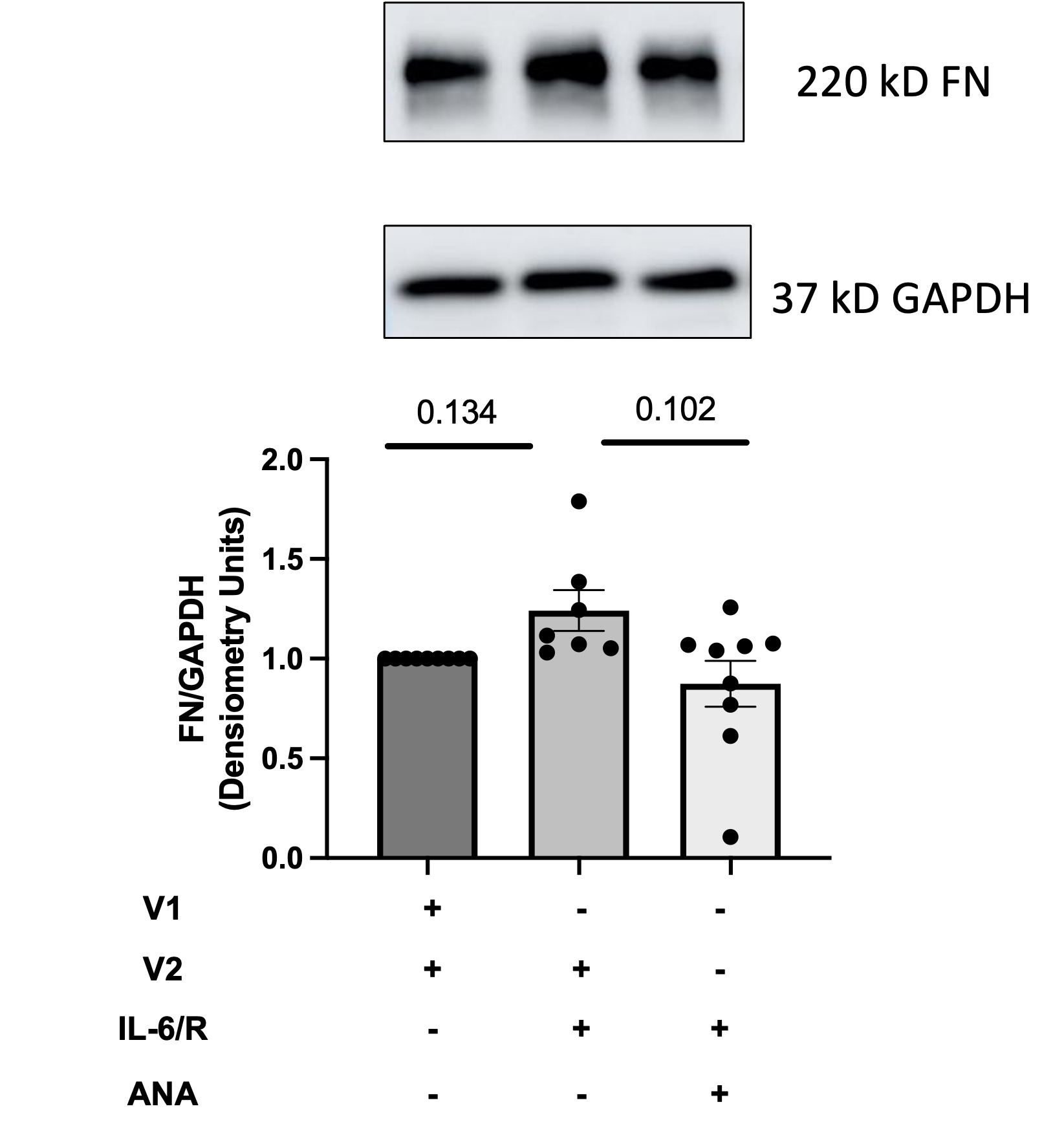

Supplement: Supplementary file 1 [file ijms-25-07227-s001.zip › supplementary figure 4.jpg]

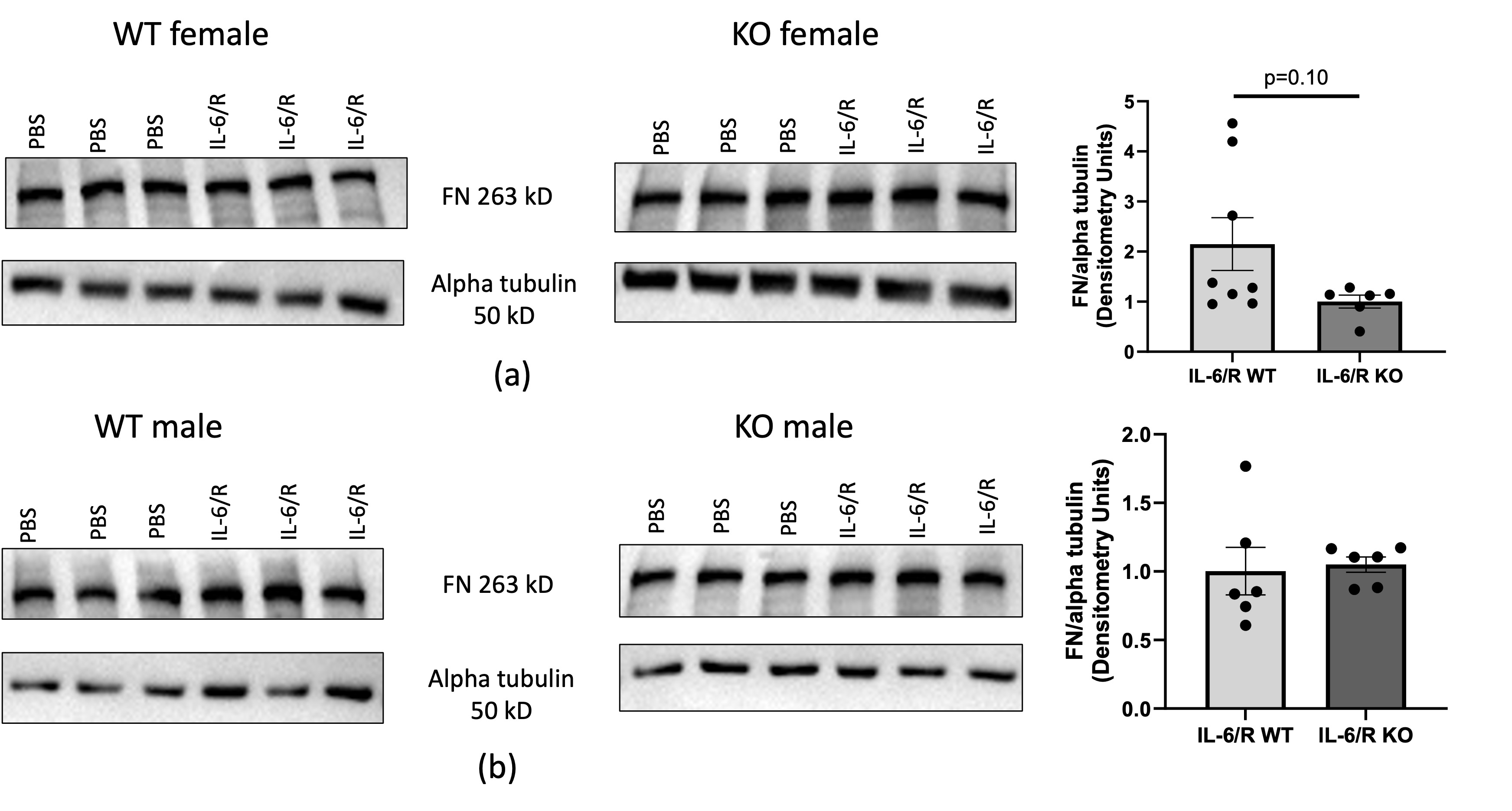

Supplement: Supplementary file 1 [file ijms-25-07227-s001.zip › supplementary figure 5.jpg]

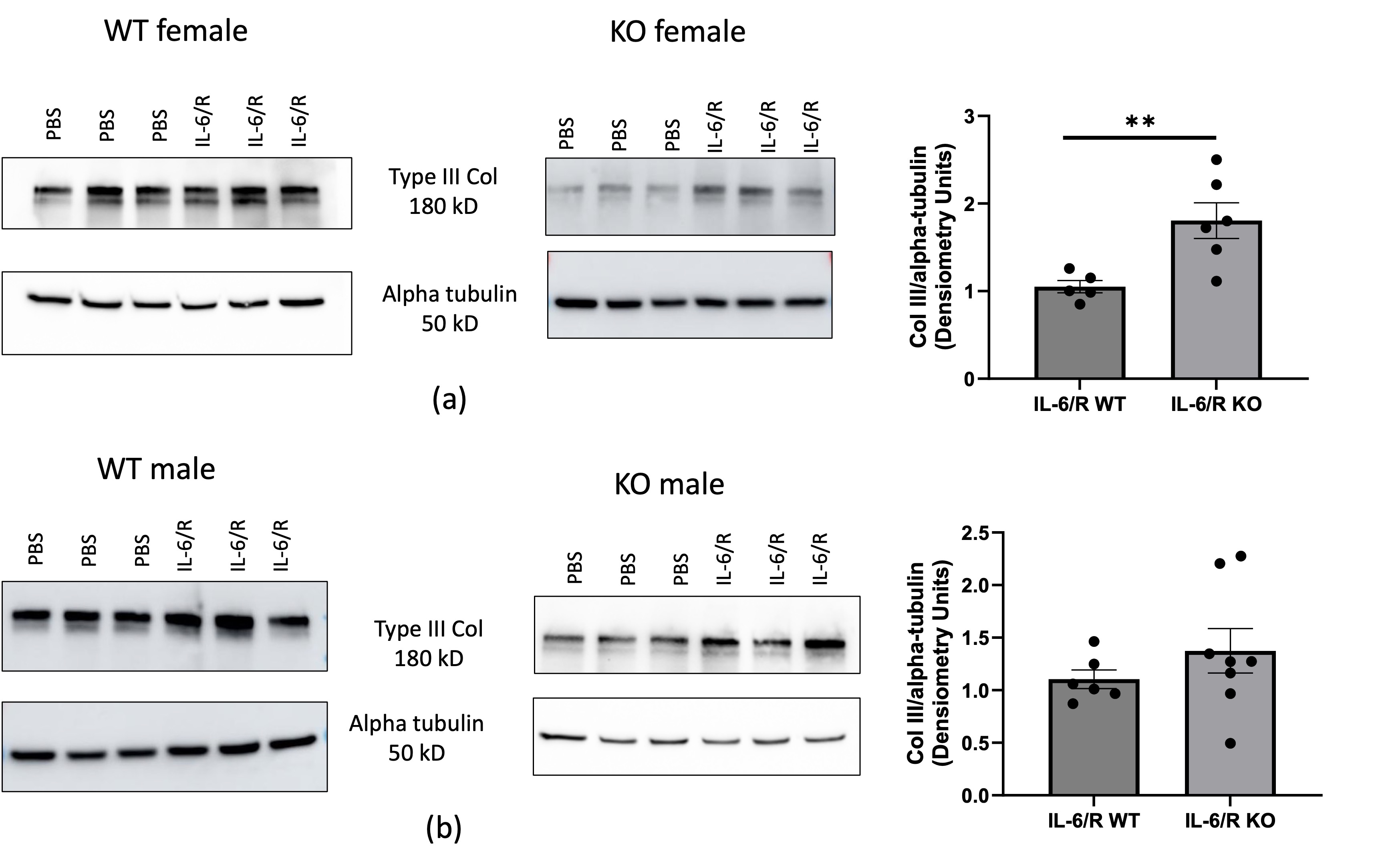

Supplement: Supplementary file 1 [file ijms-25-07227-s001.zip › supplementary figure 6.jpg]

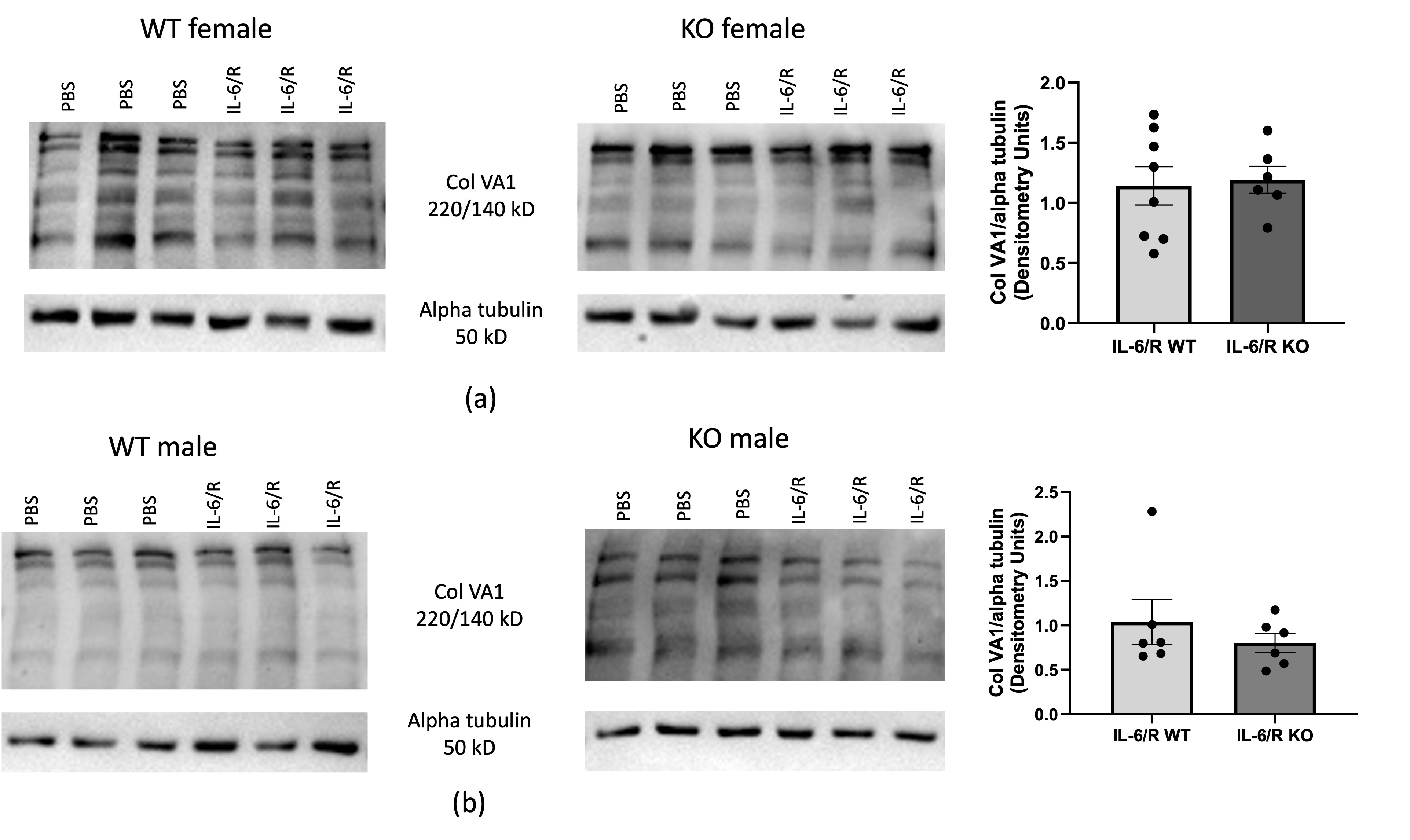

Supplement: Supplementary file 1 [file ijms-25-07227-s001.zip › supplementary figure 7.jpg]
